# Assessing the Full Burden of Respiratory Syncytial Virus in Young Infants in Low- and Middle-Income Countries: The Importance of Community Mortality Studies

**DOI:** 10.1093/cid/ciab486

**Published:** 2021-09-02

**Authors:** Padmini Srikantiah, Prachi Vora, Keith P Klugman

**Affiliations:** Pneumonia Team, Global Health Division, Bill & Melinda Gates Foundation, Seattle, Washington, USA

**Keywords:** respiratory syncytial virus, community mortality, burden

## Abstract

The Bill & Melinda Gates Foundation supported respiratory syncytial virus (RSV) mortality surveillance studies in several low- and middle-income countries to address the striking gap in community mortality burden data from these geographies. The compelling findings generated from these studies reveal a high unmeasured burden of community RSV mortality, particularly among infants aged <6 months who are the target population for RSV immunization products currently in late-stage clinical development. These findings should inform revised global RSV mortality estimates and inform policy decisions on RSV vaccine financing and prioritization at the global and national levels.

Respiratory syncytial virus (RSV) is the most common cause of severe lower respiratory tract infection (LRTI) in the first 6 months of life. The overwhelming burden of death occurs in low- and middle-income countries (LMICs) [1]. However, RSV mortality data from these geographies are sparse and, when available, generally reflect in-hospital deaths. Because of poor access to healthcare and other structural challenges, a sizable proportion of RSV-related deaths among infants in LMICs occur at home or in the community [2].

Between 2011 and 2013, a Bill & Melinda Gates Foundation–supported study in low-income neighborhoods of Buenos Aires documented striking seasonal peaks in all-cause LRTI mortality among infants in the community that consistently coincided with seasonal spikes of in-hospital RSV infant deaths, suggesting an undetected and unmeasured out-of-facility burden of RSV deaths among vulnerable communities [3]. Similar seasonal increases in community-level all-cause LRTI mortality patterns have been reported from Indonesia and Bangladesh [4]. These observations have led to adjustments to account for unmeasured community mortality in published global RSV mortality burden estimates, which indicated 27 500 annual in-hospital deaths among infants aged <6 months and up to 118 000 deaths in children aged <5 years in 2015 [4]. However, estimation of the full picture of RSV mortality burden is not possible without a more complete understanding of RSV community deaths. It is particularly important to quantify this burden among infants aged <6 months who account for the majority of RSV deaths and form the target population for maternal vaccines and prophylactic monoclonal antibodies, currently in late-stage clinical development, that hold potential to greatly reduce RSV-related mortality.

Unlike many other respiratory pathogens, detection of RSV in the nasopharynx is highly likely to indicate RSV illness. In a systematic review and analysis of 18 592 acute lower respiratory infection cases in young children across 23 studies, Shi and colleagues demonstrated that detection of RSV was significantly more common in children with acute LRTI than asymptomatic controls (odds ratio, 9.79; 95% confidence interval, 4.98–19.27), with an attributable fraction among exposed of 90% [5]. Similar findings were noted in the multicountry Pneumonia Etiology Research for Child Health study, where detection of RSV was also strongly associated with acute LRTI in cases with pneumonia compared with age-matched community controls [6].

In the Pneumonia Program at the Gates Foundation, we leveraged these important findings to support RSV community mortality surveillance studies in Argentina, India, Pakistan, and Zambia, representing diverse LMIC geographies. All sites focused on the collection of nasopharyngeal (NP) swabs from recently deceased infants to assess the presence of RSV by polymerase chain reaction. Two of the studies, in Zambia and Argentina, enrolled deceased infants at the morgue, while the teams in India and Pakistan each implemented active surveillance to prospectively identify infant deaths within a defined community. The studies in Zambia and Pakistan focused on infants aged <6 months, while those in Argentina and India included those aged <5 years and <2 years, respectively. Common to all 4 studies was a goal of generating evidence on the burden of RSV among community deaths in young infants (aged <6 months) in LMICs.

In Lusaka, Zambia, the study team made use of local regulations mandating medical certification of all deaths before release for burial, thus enabling the capture of the majority (>80%) of deaths in the study catchment area. Community deaths accounted for 70% of infants enrolled. Over the course of 3 years, nearly 800 infants (aged <6 months) who died in the community were enrolled on presentation to the morgue; RSV was detected in nearly 8% (*Murphy et al, CID 2021, current issue).* Investigators in Pakistan used community-based active mortality surveillance for infants aged <6 months in 4 low-income settlement areas in peri-urban Karachi (*Kazi et al, CID 2021, current issue*). Half of all deaths occurred in the community, though RSV detection during the surveillance period was lower (3.7%).

In central India, the study was conducted in Melghat, a rural area with high baseline infant mortality (>50/1000 live births), where the team used prospective active weekly surveillance for LRTIs and deaths across 93 villages. RSV was detected in 7.3% of annual post-neonatal mortality, and 80% of RSV deaths occurred in the community. In India, their active surveillance platform enabled calculation of a population-based estimate of an RSV LRTI mortality rate and indicated that mortality was 7- to 10-fold higher in the community compared with the hospital (*Simoes et al, CID 2021, current issue*). This is notably higher than the inflation factor of 2.2 applied to adjust RSV LRTI mortality in children aged <5 years in LMICs to account for increased unmeasured mortality in community settings in currently published global RSV mortality estimates [4] and points to the need to account for these emerging data as well as for this younger age group when revising these estimates.

In Argentina, investigators partnered with the judicial morgue serving impoverished districts surrounding Buenos Aires to evaluate RSV in community deaths among children aged <5 years. Early study data from 2016–2017 indicated detection of RSV in almost 10% of these children, mostly among infants aged <12 months [7]. In the current issue, the investigators report on their expanded evaluation of RSV community mortality in 2019 in the same neighborhoods where, in addition to an NP swab, they collected minimally invasive tissue samples (MITS) from multiple organs, used molecular methods to assess for 16 additional pathogens, evaluated pathologic findings, and assigned cause of death attribution using methods standardized in the Child Health and Mortality Prevention Surveillance (CHAMPS) platform (*Caballero et al, CID 2021, current issue*). Among 63 community deaths of children aged <5 years, RSV was detected in 12 (19%). All RSV-positive deaths were in infants aged <6 months (12 of 44, 27%), and RSV was deemed to be in the causal chain of death in 11 (91%).

In all 4 settings, infants aged 0–3 months accounted for most RSV community deaths. In fact, relative younger age at death was consistently associated with community RSV deaths across RSV GOLD’s 38 country comparison of community vs hospital deaths (*Mazur* et al*, CID 2021, current issue*). This is important because it is exactly this younger infant age group that stands to benefit most from RSV immunization products, where the highest efficacy is likely to be seen in the first 90 days of life. These community findings have important implications for the potential impact that RSV prophylaxis may have on mortality and are important to drive immunization policy.

In addition to these 4 studies, all reported in this supplement, the CHAMPS platform permitted assessment of multiple etiologies of illness and death among deceased infants and children using molecular and pathologic evaluation of biological and MITS specimens from multiple organs, as well as assignment of the causal chain for each death using a standardized process. Although the majority of those evaluated in CHAMPS were facility deaths, these comprehensive data from sites in 7 African and South Asian countries provide additional important insights into the role of RSV in early infant mortality. Most notably, RSV was detected in 12% of deaths among infants aged 28 days–6 months and was determined to be in the causal chain of 6.5% of all deaths in this age group (*Blau et al, CID 2021, current issue*).

Two key limitations of the community mortality studies in Zambia, India, and Pakistan are readily acknowledged: attribution of RSV as a cause of death based only on an NP specimen and the lack of testing for other pathogens. In Argentina, where MITS, multiple pathogen testing, and a standardized causal attribution process were applied in a community mortality surveillance platform, RSV was assigned to be in the causal chain in most deaths where it was detected. This was different in CHAMPS, where in the post-neonatal age group (28 days–6 months), RSV was determined to be in the causal chain in roughly 50% of RSV-positive deaths, and very few deaths were deemed to be due to only RSV. This may be, in part, a reflection of greater burden of additional infectious disease etiologies, including human immunodeficiency virus and malaria, in the higher mortality settings that characterize CHAMPS sites, as well as the greater complexity of facility-based deaths with multiple comorbidities. Nevertheless, it is important to recognize that RSV can increase risk for both bacterial coinfection as well as for subsequent LRTI episodes and thus serve as a triggering event for a poor outcome [8, 9]. Furthermore, all of these studies assessed the presence of RSV at a single point in time. A recent analysis suggested that there may be an increased risk of death in the first month after an RSV illness episode [10]. Thus, RSV may go undetected at the time of death but still have played a role toward the demise of an infant, suggesting that attribution of RSV even in the current studies may represent an underestimation of true mortality burden.

Measuring community mortality is a significant and challenging undertaking. Critically important to the success of all 4 studies was the importance of thoughtfully engaging the community to identify, enroll, and collect specimens from recently deceased infants in a timely fashion. While the nature of this engagement differed in each setting, the need to develop culturally sensitive approaches to meeting with families soon after the death of their child was universal. Investigators developed locally appropriate strategies that considered religious and cultural beliefs and practices to provide grief counseling to family members at the time of enrollment and beyond. Consistent engagement with local leaders was similarly critical to ensuring community acceptance of surveillance efforts.

The compelling data generated from these surveillance efforts have revealed a high and previously unmeasured burden of community RSV mortality among young infants in LMICs. These findings are critical to enabling a more accurate understanding of the preventable burden of RSV deaths and should be incorporated into revised global RSV mortality estimates. RSV maternal vaccines and infant monoclonal antibody prophylaxis are currently in phase 3 trials and, if found to be efficacious, could be licensed within the next few years. It is imperative that decisions on global immunization financing as well country-level prioritization on vaccine preventable diseases be informed by estimates that reflect the complete burden of RSV.

## Notes

***Financial support.*** This work was supported by the Bill & Melinda Gates Foundation.

***Supplement sponsorship.*** This supplement is sponsored by the Bill and Melinda Gates Foundation.

***Potential conflicts of interests.*** All authors are employed by the Bill & Melinda Gates Foundation, which funded the studies described in this supplement. All authors have submitted the ICMJE Form for Disclosure of Potential Conflicts of Interest. Conflicts that the editors consider relevant to the content of the manuscript have been disclosed.
